# Generalized Tetanus Initially Presenting with Dysmasesis

**DOI:** 10.7759/cureus.644

**Published:** 2016-06-17

**Authors:** Parvaiz M Zunga, Shah Faisal Ahmad Tarfarosh, Omar Farooq, Ishrat H Dar, Samia Rashid, Ummer Yaseen

**Affiliations:** 1 Registrar, SMHS Hospital, Srinagar; 2 MBBS, Acharya Shri Chander College of Medical Sciences and Hospital, Jammu, J & K, India; 3 Assistant Professor, SMHS Hospital, Srinagar; 4 Professor and Head of Unit, SMHS Hospital, Srinagar

**Keywords:** tetanus, dysmasesis, trismus, stiffness, neurology

## Abstract

A 58-year-old male farmer, with no significant history of any chronic morbidity, was admitted via the Emergency Department of SMHS Hospital, Srinagar, with a history of an injury to the hand 20 days earlier followed by a three-day history of dysmasesis (difficulty chewing), progressing to trismus and generalized stiffness interfering with his daily activities. The patient was clinically managed as tetanus on grounds of high clinical suspicion. The patient was treated for a week and discharged without any sequelae to follow-up in the Neurology Outpatient Department of the SMHS Hospital and is currently doing well. After a week of successful management, we received the blood and wound culture reports of the patient that had been sent at the time of his admission to the hospital, which overwhelmingly tested positive for *Clostridium tetani*. Tetanus is a disease to be suspected post-trauma in patients, especially in developing countries like India. Despite active and passive immunization, it continues to be a significant public health problem in developing countries and should be readily suspected and treated. Although prevention is important for tetanus, the outcomes can be improved by early clinical diagnosis and treatment.

## Introduction

Tetanus is an infectious disease caused by *Clostridium tetani,* an obligate anaerobic gram-positive bacterium, and mainly involves the nervous system with severe neurological manifestations [1]. The spores of this organism are located in soil and in the feces of humans and animals [1]. Despite the availability of passive immunization since 1893 and an effective active vaccination since 1923, tetanus remains a major health problem in the developing world and is still encountered in the developed world [1].

However, in the developed nations like the United Kingdom (UK) and United States of America (USA), the prevalence is very low. This can be acknowledged by the fact that only seven cases of tetanus were reported in the UK in the year 2013. The reporting percentage of tetanus is also low (< 10% of total cases are reported) and the worldwide incidence of tetanus is unknown. Less than 50% of the tetanus cases are reported in the USA to the Centers for Disease Control [2]. We report a rare case of generalized tetanus initially presenting with dysmasesis.

## Case presentation

A 58-year-old normotensive, non-diabetic, nonsmoker male farmer was brought to the Emergency Department of SMHS hospital, Srinagar, by his wife with a three-day history dysmasesis (difficulty in chewing) followed by severe stiffness of jaw. It was gradual in onset and progressive. This was followed by stiffness in the upper and lower limbs. The patient could not flex his limbs due to stiffness. The patient was not able to walk due to stiffness in the lower limbs and gradually felt stiffness in the whole body over a period of three days. There was a two-day history of occasional painful spasms in the upper and lower limbs. Also, there was a history of several episodes of profuse sweating.

There was a history of trauma to the right hand (palm), which the patient sustained while working in fields about 20 days earlier; unfortunately, he neglected the wound and did not apply any dressing over the wound. No history of any visual impairment, doubling of vision, any inward or outward deviation of the eyes, any facial asymmetry, deviation of the angle of the mouth, drooling of saliva, or hearing impairment was present. The patient’s history was also negative for any difficulty in swallowing, nasal twang of voice, nasal regurgitation, difficulty in breathing, muscle twitching, paresthesias, numbness of any part of the body, inability to feel hot or cold, bladder and bowel involvement, any rash, oral ulcers, joint pains, any swelling/redness/tenderness of the joints, or photosensitivity.

**Figure 1:**
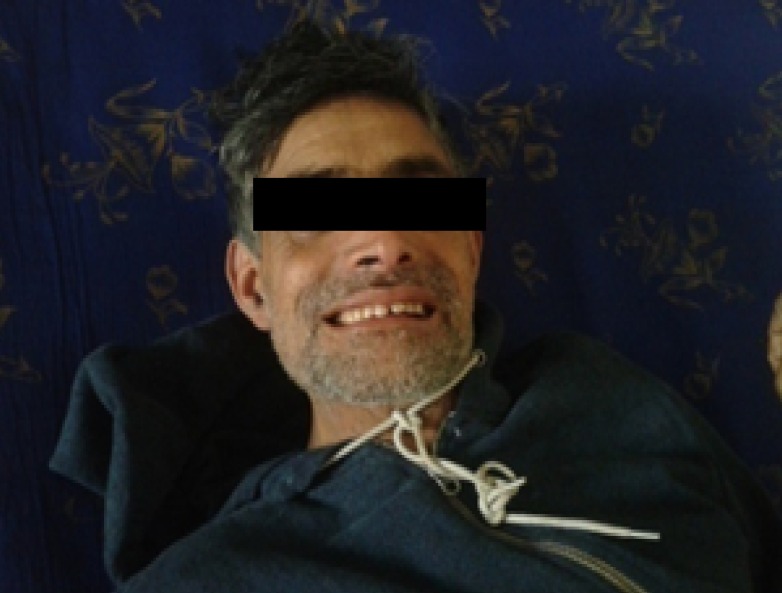
Risus sardonicus in the patient of generalized tetanus

On examination, the patient was conscious and oriented to time, place, and person. Pulse rate was 88 beats per minute and it was regular. The patient’s blood pressure was recorded as 120/80 mmHg, the temperature was 98˚F (axillary), and respiratory rate was 16 breaths per min. There was no pallor, icterus, cyanosis, or edema. The jugular venous pressure was not raised, and there was no thyromegaly. Risus sardonicus was present as shown in Figure 1. Informed patient consent was obtained for treatment.

Cardiorespiratory findings were insignificant for any abnormality. However, on abdominal examination, board-like rigidity was felt and the recti abdominis were palpable, but no organomegaly or ascites was found.

**Figure 2:**
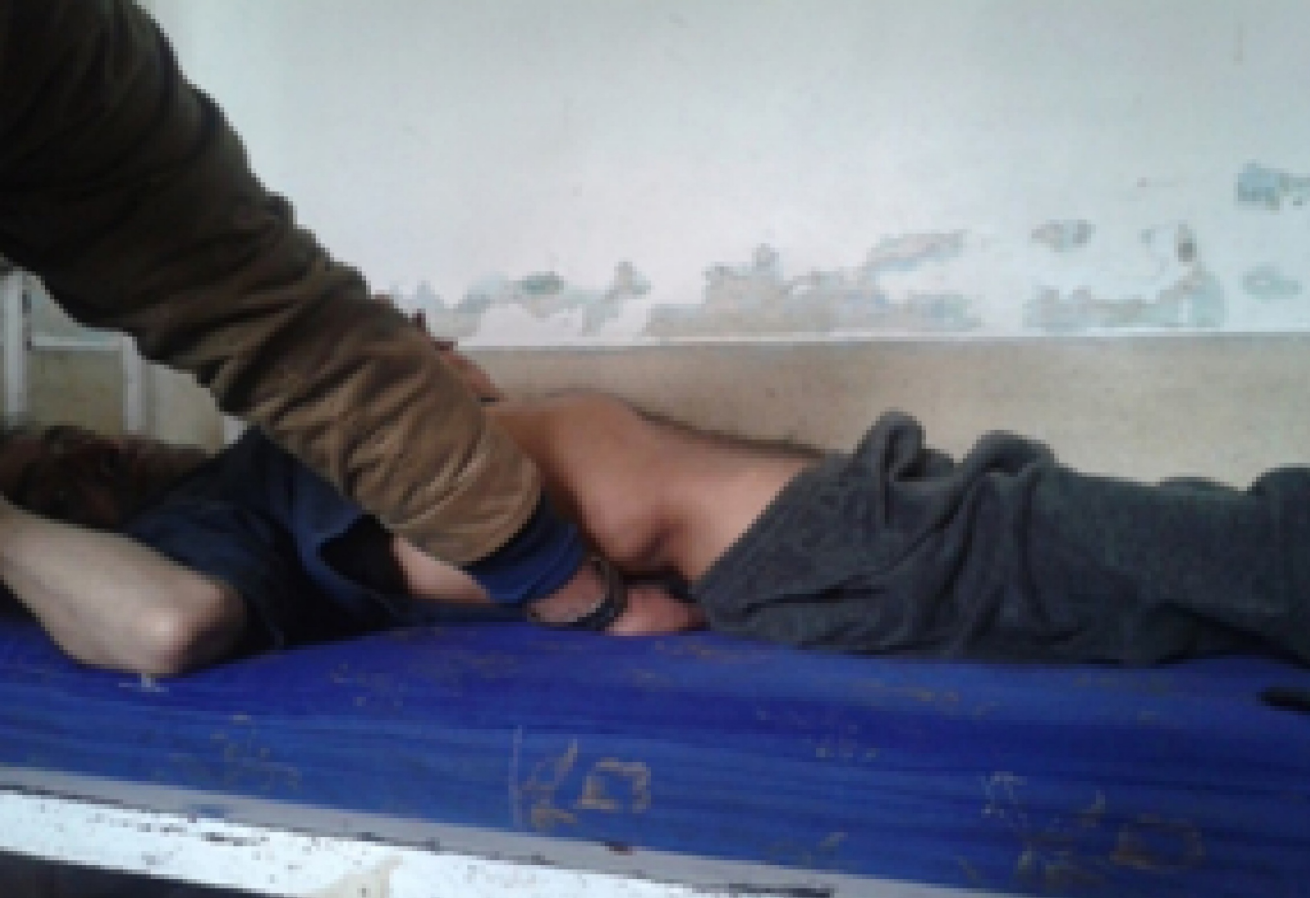
Opisthotonus in the patient of generalized tetanus

On central nervous system examination, higher mental functions were normal, speech was normal, MMSE score was 26/26, and muscle bulk was normal (equal bilaterally). Both the upper and lower limb spasticity was Grade 4. Opisthotonus was present as seen in Figure 2. Strength could not be assessed due to generalized spasticity. Deep tendon reflexes were exaggerated (3+). Babiniski’s sign was positive. There were no cerebellar signs. Spine examination was normal. Sensory examination was also normal.

All the biochemical parameters, including complete blood count, renal function tests, and liver function tests, were normal at the time of presentation. Arterial blood gas analysis was also normal. Serum Ca^2+^ and PO^4+^ levels were normal. Routine urine examination was normal. Creatine phosphokinase levels were within normal limits. Blood and wound culture specimens were sent at the time of admission, but results were not received until a week after starting the treatment; the results were positive for the growth of rod-shaped, gram-positive *Clostridium tetani*. Chest x-ray and ECG were normal. Magnetic resonance imaging (MRI) of the brain was normal. Serum anti-tetanus immunoglobulin (IgG antibodies) were low at 0.04 IU/L, and the immunization history was not remembered by the patient or his attendant.

The patient was managed as a case of tetanus on high clinical suspicion. Our management modalities included nasogastric tube feeding, antiseptic dressing, and injection of human anti-tetanus globulin (3,000 units) intramuscular stat and injection tetanus toxoid intramuscular stat. The patient was then put on all of the following medications for seven days: injections of Metrogel (metronidazole), 500 mg intravenous q.i.d., diazepam tablets, 10 mg q.i.d., injections of heparin, 5,000 units subcutaneous b.i.d., and Liofen xl (Baclofen) tablets, 5 mg t.i.d. for two days, 10 mg t.i.d. for two days, 15 mg t.i.d. for 2 days, and 20 mg t.i.d. on the last day of hospitalization.

Within a week, the patient showed marked improvement, the dysmasesis, trismus, and stiffness improved significantly, and he was able to continue his daily activities without any help. The patient was discharged to follow-up in the Neurology OPD at our hospital. On discharge, diazepam, 10 mg tablets t.i.d., and Liofen xl (Baclofen), 20 mg tablets b.i.d. were prescribed for one week.

## Discussion

The word ‘tetanus’ is believed to be derived from the Greek word ‘tetanos’, which means “to contract”. Some of the clinical features of tetanus were known to man from ancient times. Tetanus-like features were described in the Ancient Egyptian “Edwin Smith Papyrus”, which dates back to 1600 BC. Also, the features of generalized tetanus, as described in our medical textbooks and references, are found superficially mentioned in the writings of Hippocrates (400 BC, Ancient Greece) and in the Ayurveda texts (400 AD, Ancient India) [2]. *Clostridium tetani*, a rod-shaped, obligate anaerobic gram-positive bacterium, is widely present in the soil. The main mode of infection is through open wounds. Many cases of tetanus caused by minor traumas, like gardening, minor injuries in farm fields, battlefield injuries, and tooth extraction using unsterilized equipment, have been reported [3]. Neonatal tetanus occurs in the newborn around the first week of life [4]. It is impossible to predict the development of tetanus from the severity of the trauma. However, it is possible to reduce the incidence by wound irrigation and debridement of the injured wounds because *C. tetani* mainly adhere to necrotizing tissue [1]. It is difficult to identify or isolate *Clostridium* *tetani*, but it can be readily diagnosed by bacterial culture or serological testing, which, however, is not necessary for diagnosis [5-6]. Even if the bacterium can be isolated, several days are required to obtain the results [1].

Thus, when symptoms of tetanus-like dysmasesis, trismus, rigidity, and systemic convulsions develop following trauma, tetanus should be actively suspected and rapidly started on appropriate treatment. Because our patient was admitted after tetanus was diagnosed on just his physical examination and history alone and then treated relatively early, he was able to resume his life with no serious complications or sequelae. The diagnosis was suspected on clinical grounds with a significant history of trauma followed by dysmasesis, trismus, generalized rigidity, and tonic posturing (opisthotonus). He was actively treated and discharged from the hospital without any sequelae and is on follow-up. Therefore, it is imperative to have a high clinical suspicion of tetanus, especially in developing countries like India, and to institute early diagnosis and treatment in order to avoid permanent damage and to get the best patient results. Treatment should be empirically instituted, pending test results. If clinicians pay attention to the possibility of tetanus development, treatment can be rapidly initiated.

## Conclusions

We encountered a patient in whom tetanus developed after an injury to his hand in a farm accident and was actively treated and discharged without any sequelae. Although prevention is important for tetanus, the outcomes can be improved by early clinical diagnosis and treatment.
