# Correction: Generalized Tetanus Initially Presenting with Dysmasesis 

**DOI:** 10.7759/cureus.c4

**Published:** 2016-09-19

**Authors:** Parvaiz M Zunga, Shah Faisal Ahmad Tarfarosh, Omar Farooq, Ishrat H Dar, Samia Rashid, Ummer Yaseen

**Affiliations:** 1 Registrar, SMHS Hospital, Srinagar; 2 MBBS, Acharya Shri Chander College of Medical Sciences and Hospital, Jammu, J & K, India; 3 Assistant Professor, SMHS Hospital, Srinagar; 4 Professor and Head of Unit, SMHS Hospital, Srinagar

**Title:** Generalized Tetanus Initially Presenting with Dysmasesis

**Authors:** Shah Faisal Ahmad Tarfarosh, Omar Farooq, Ishrat H. Dar, Samia Rashid, Ummer Yaseen

**Original Citation:** Zunga P M, Tarfarosh S, Farooq O, et al. (June 17, 2016) Generalized Tetanus Initially Presenting with Dysmasesis . Cureus 8(6): e644. doi:10.7759/cureus.644

The authors were originally placed in the incorrect order and Ummer Yaseen was erroneously added as an author instead of Mohd Iqbal Dar. The author list should be corrected as follows.

**Corrected Author List: **Omar Farooq, Ishrat H. Dar, Mohd Iqbal Dar, Samia Rashid, Parvaiz M. Zunga, Shah Faisal Ahmad Tarfarosh

